# Supplementary material for: Does “lottery culture” affect household financial decisions? Evidence from China
Source: PLoS One. 2022 Oct 14;17(10):e0275717. doi: 10.1371/journal.pone.0275717 (PMC9565457; doi:10.1371/journal.pone.0275717)
Supplement: Attachment — Submitted filename: Review opinions.doc [file pone.0275717.s001.doc]

**审稿意见：大修**

问题1：“彩票文化”（来自CNRDS的数据）是否可以具体到每个省的家庭?能否与CHFS的家庭数据相吻合?请作者给出答案。

建议1：本文使用是横截面数据，所以不应出现地区固定效应（因为本文不是面板数据），所以建议作者重新验证实证结果，把“Area fixed effects”这一行去掉，以避免犯常识性实证错误。

建议2：建议本文增设变量介绍，分别列明1）被解释变量；2）核心解释变量；3）控制变量，以便更具清晰性，而不应该杂糅在模型表达式和数据介绍中，显得杂乱无章。

建议3：第3个被解释变量——the diversity of financial portfolio.，衡量方法公式有误，认为不应是$D\text{iversity}=1\text{-}\sum_{\text{i}=1}^{N} W_{\text{i}}^{2}$，而应该是$D\text{iversity}=\sum_{\text{i}=1}^{N} W_{\text{i}}^{2}$，这样表示的是金融组合多样性指数。

建议4：建议本文的3个被解释变量，在变量介绍中，提供变量缩写，以便与后文的实证结果相吻合，不至于让人感觉很突兀。

建议5：建议实证结果中的所有控制变量不用进行实证结果解释分析，并且在实证结果表格中，不用列出具体的回归系数和标准误，用YES表示控制即可，以突出核心解释变量对被解释变量的影响，呼应主题。

建议6：关于内生性的说明中，建议就选择的工具变量，对其满足的两个基本条件——外生性和相关性条件进行详细说明解释（因为本文只解释了相关性，没有解释外生性）；对于使用的工具变量法，应进行弱工具变量检验和工具变量外生性检验（本文只说明解释了弱工具变量检验），建议作者对此进行补充检验，以便保证本文内生性说明的完整性。

建议7：稳健性检验，对于前2个被解释变量，本文使用logit模型替换OLS模型，第三个被解释变量——the diversity of financial portfolio.，本文使用什么具体变量来替换第三个被解释变量，请作者在文中解释清楚（表4，第5列），并且建议表4中第3—4列应该去掉，因为和本文稳健性检验没有任何关系。

建议8：进一步分析中，即本文的机制检验，公式（3）和（4）中的随机扰动项应该用不同的字母表示，以进行相应区分；并且表格中所有控制变量不用列出具体的回归系数和标准误，用YES表示控制即可。

建议9：建议在研究假设中，加入机制分析的相应研究假设（假设3a：“彩票文化”通过家庭风险态度影响家庭财务决策”；假设3b：“彩票文化”通过人力资本影响家庭财务决策。），以便前后文呼应。

建议10：注意修正文中翻译错误（如题目Does “Lottery Culture” Affect Household Financial Decisions?—— Evidence from China；Previous research show that “lottery culture” have significant impacts on corporate decisions, household investment portfolio choices and stock returns. Chen et al. [2]等等）和规范本文参考文献格式。

Review comments: ***major revision***

**Question 1:** Can the "lottery culture" of each province from CNRDS be specific to families? Can it be consistent with the family data of CHFS? Please the authors give the answer.

**Suggestion 1:** This paper uses cross-sectional data, so there should be no “area fixed effects” (because this paper is not panel data). Therefore, it is suggested that the author revalidate the empirical results and remove the line "Area fixed effects" to avoid common-sense empirical errors.

**Suggestion 2:** It is suggested to add “variable introduction” in this paper, listing “(1) explained variables; (2) Core explanatory variables; (3) Control variables” for more clarity, and should not be mixed into model expressions and data introductions, which appear to be chaotic.

**Suggestion 3:** The third explained variable —— “the diversity of financial portfolio”. The measurement formula is wrong. It is believed that it should not be “$D\text{iversity}=1\text{-}\sum_{\text{i}=1}^{N} W_{\text{i}}^{2}$”, but should be “$D\text{iversity}=\sum_{\text{i}=1}^{N} W_{\text{i}}^{2}$”，So this is the financial portfolio diversity index..

**Suggestion 4:** It is suggested that the three explained variables in this paper should be abbreviated in the introduction of variables, so as to be consistent with the empirical results in the following article and not make people feel very abrupt.

**Suggestion 5:** It is suggested that all control variables in the empirical results do not need to be analyzed by empirical results interpretation. In addition, specific regression coefficients and standard errors are not required to be listed in the table of empirical results, and “YES” can be used to represent control, so as to highlight the influence of the core explanatory variables on the explained variables and echo the theme.

**Suggestion 6:** In the explanation of endogeneity, it is suggested to explain in detail the two basic conditions —— exogeneity and correlation conditions —— for the selected instrumental variables (because this paper only explains correlation, not exogeneity);For the instrumental variables method used, the test for weak instrumental variables and the test for exogeneity of instrumental variables (only the weak instrumental variables test is explained in this paper) should be performed, and it is suggested that the authors conduct additional tests to ensure the completeness of the endogeneity explanation in this paper.

**Suggestion 7:** Robustness test: For the first two explained variables, this paper uses the logit model to replace the OLS model. For the third explained variable —— the diversity of financial portfolio. Please explain clearly what specific variable is used to replace the third explained variable in this paper (Table 4,Column 5), and suggest that columns 3-4 of Table 4 should be dropped because they have nothing to do with the robustness check for this article.

**Suggestion 8:** In the further analysis, that is, the mechanism test in this paper, the random disturbance terms in equations (3) and (4) should be represented by different letters to distinguish them accordingly; In addition, there is no need to list specific regression coefficients and standard errors for all control variables in the table, and “YES” can be used to represent control.

**Suggestion 9:** It is suggested to add the corresponding research hypothesis of mechanism analysis into the research hypothesis (Hypothesis 3a: "Lottery culture" influences household financial decisions through household risk attitude "; Hypothesis 3b: "Lottery culture" influences household financial decisions through human capital.) In order to echo before and after the part.

**Suggestion 10:** Pay attention to the translation errors in the text (e.g., Does "Lottery Culture" Affect Household Financial Decisions?***——*** Evidence from China; Previous research show that "lottery culture" ***have*** significant impacts on corporate decisions, Household investment portfolio choices and stock returns. Chen et al.[2], etc.) and standardize the reference format of this paper.
