# Supplementary material for: Does “lottery culture” affect household financial decisions? Evidence from China
Source: PLoS One. 2022 Oct 14;17(10):e0275717. doi: 10.1371/journal.pone.0275717 (PMC9565457; doi:10.1371/journal.pone.0275717)
Supplement: Attachment — Submitted filename: Report on the Manuscript Number PONE-D-22-15747.docx [file pone.0275717.s002.docx]

Report on the manuscript entitled “Does ‘Lottery Culture’ Affect Household Financial Decisions? Evidence from China” by the Dan Wang

The authors analyze the relationship between “lottery culture” and family financial decision-making through cross-sectional data, and make an analysis of the intermediary mechanism, which is well organized.

However, some issues still need to be improved:

(1) What's your definition of “lottery culture”? Can it comprehensively be measured by the ratio of the per capita lottery sales to the per capita GDP of provinces? My suggestion is that the authors should cite more literature.

(2) Is the result of analysis with cross-sectional data convincing? Why didn’t you use panel data? Please explain why you used cross-sectional data instead of panel data.

(3) There are many factors that affect family financial decision-making. Please explain the selection of control variables and the possible reasons for the analysis results.

(4) Is there a science to choosing the number of Chinese Football Super League and China Basketball Association teams as the instrument variable? Please explain it by citing more literature.

(5) Is there any theoretical support for the intermediary mechanism? Why didn’t you propose the hypotheses on it in part 2 (Hypotheses Development)? My suggestion is that the authors should add some description about mediated relation between “lottery culture” and family financial decision-making in the second part.

(6) Is the conclusion of the data analysis in 2017 applicable after 2020 (COVID-19)?
