# Supplementary material for: Does “lottery culture” affect household financial decisions? Evidence from China
Source: PLoS One. 2022 Oct 14;17(10):e0275717. doi: 10.1371/journal.pone.0275717 (PMC9565457; doi:10.1371/journal.pone.0275717)
Supplement: Attachment — Submitted filename: Response to Reviewers.doc [file pone.0275717.s003.doc]

**Response to Reviewer 1**

**Note:** In the review response that follows, we use the font **Arial** to indicate the reviewer’s comments and the font **Times New Roman** to indicate our responses.

**审稿意见：大修**

问题1：“彩票文化”（来自CNRDS的数据）是否可以具体到每个省的家庭?能否与CHFS的家庭数据相吻合?请作者给出答案。

回答1：非常感谢审稿专家的问题。本文参考赵等（2018）的做法，通过人均彩票销售额与各省人均GDP之比的1000倍得到的指数来衡量 “彩票文化”这一个变量。这是一个指数，表征一个省份的“彩票文化”，本文将这一指数通过CHFS数据库中的家庭所在地进行匹配，如果这个家庭属于这个省份，那就用这个省份的“彩票文化指数”来表征这个家庭的“彩票文化”。原本是想做到市一级的数据，但是由于市级层面的彩票销售额相关数据没有公布，没办法去计算市级层面的彩票文化指数。

参考文献：Zhao Q, Zhao W, Lu D, Zhao Q. Lottery and enterprise innovation: A research based on the view of culture. Finance & Trade Economics. 2018; 39:122–40.

Christensen D M，Jones K L, Kenchinton D G. Gambling attitudes and financial misreporting. Contemporary Accounting Research,2018,35(3) ;1229—1261

建议1：本文使用是横截面数据，所以不应出现地区固定效应（因为本文不是面板数据），所以建议作者重新验证实证结果，把“Area fixed effects”这一行去掉，以避免犯常识性实证错误。

回应1：感谢审稿专家的意见。在公开发表的期刊中，不乏有在截面数据中控制行业，城市，省份，经济区域固定效应的研究。比如孙伟增“空气污染与劳动力的空间流动”2019年发表在经济研究上的这篇文章。研究设计里（Y:劳动力是否流动；X:pm2.5）在控制变量的基础上进一步增加了城市固定效应，缓解了可能由城市控制变量遗漏导致的内生性问题，得到了更为稳健的估计结果。

本文使用的“Area fixed effects”和解释变量被解释变量的区域不是一个概念，这里使用的是“东部地区、东北地区、中部地区和西部地区”。中国政府根据区域经济和社会发展水平将各省划分为四个经济区：东部地区、东北地区、中部地区和西部地区。因此，本文还将经济区固定效应纳入回归，以控制区域差异，缓解了可能由区域控制变量遗漏导致的内生性问题，得到了更为稳健的估计结果。

建议2：建议本文增设变量介绍，分别列明1）被解释变量；2）核心解释变量；3）控制变量，以便更具清晰性，而不应该杂糅在模型表达式和数据介绍中，显得杂乱无章。

回应2：感谢审稿专家指导性建议。本文在 “3.2 数据描述”部分增加了“3.2.1被解释变量”、“3.2.2核心解释变量”、“3.2.3控制变量”这样的标题，便于清晰看到。

建议3：第3个被解释变量——the diversity of financial portfolio.，衡量方法公式有误，认为不应是，而应该是，这样表示的是金融组合多样性指数。

回应3：非常感谢审稿专家的耐心指正。我们在查阅文献后，对存在的错误进行了更正。

建议4：建议本文的3个被解释变量，在变量介绍中，提供变量缩写，以便与后文的实证结果相吻合，不至于让人感觉很突兀。

回应4：感谢审稿专家的建议。为了便于后续更加清晰的阅读实证结果，下文的图表将用Formal表示Formal Financial Market Participation，用Informal表示Informal Financial Market Participation，用Diversity表示Diversity of Financial Portfolio.

建议5：建议实证结果中的所有控制变量不用进行实证结果解释分析，并且在实证结果表格中，不用列出具体的回归系数和标准误，用YES表示控制即可，以突出核心解释变量对被解释变量的影响，呼应主题。

回应5：感谢审稿专家的指导性建议。根据意见，控制变量用YES表示控制，删除了对所有控制变量的实证结果解释分析。

建议6：关于内生性的说明中，建议就选择的工具变量，对其满足的两个基本条件——外生性和相关性条件进行详细说明解释（因为本文只解释了相关性，没有解释外生性）；对于使用的工具变量法，应进行弱工具变量检验和工具变量外生性检验（本文只说明解释了弱工具变量检验），建议作者对此进行补充检验，以便保证本文内生性说明的完整性。

回应6：感谢审稿专家的建议。已在论文里做了相关的补充：无论在理论上还是现实中，家庭金融市场参与都不会受到体育赛事的影响，这足以表明工具变量的外生性。为了更严格地解决内生问题，我们进行第二阶段回归，并将残差回归到IV。如果p值大于0.1且原始假设未被拒绝，则IV是外生的。

建议7：稳健性检验，对于前2个被解释变量，本文使用logit模型替换OLS模型，第三个被解释变量——the diversity of financial portfolio.，本文使用什么具体变量来替换第三个被解释变量，请作者在文中解释清楚（表4，第5列），并且建议表4中第3—4列应该去掉，因为和本文稳健性检验没有任何关系。

回应7：感谢审稿专家的建议。本文是使用家庭金融资产的类别来衡量家庭金融投资组合的多样性，如家庭金融投资组合有[债券](https://baike.baidu.com/item/债券?fromModule=lemma_inlink)、[股票](https://baike.baidu.com/item/股票?fromModule=lemma_inlink)、[投资基金](https://baike.baidu.com/item/投资基金?fromModule=lemma_inlink)，那对家庭金融资产的类别赋值为3,以此类推。表4有第3-4列，是因为本文希望将稳健性检验结果和原实证结果做一个对比，删除也没有问题。根据评审意见，做删除处理。

建议8：进一步分析中，即本文的机制检验，公式（3）和（4）中的随机扰动项应该用不同的字母表示，以进行相应区分；并且表格中所有控制变量不用列出具体的回归系数和标准误，用YES表示控制即可。

回应8：感谢审稿专家的建议。根据评审意见，已用ε代替μ，控制变量用YES表示控制。

建议9：建议在研究假设中，加入机制分析的相应研究假设（假设3a：“彩票文化”通过家庭风险态度影响家庭财务决策”；假设3b：“彩票文化”通过人力资本影响家庭财务决策。），以便前后文呼应。

回应9：已补充3.2 Mechanism analysis。

建议10：注意修正文中翻译错误（如题目Does “Lottery Culture” Affect Household Financial Decisions?—— Evidence from China; Previous research show that “lottery culture” have significant impacts on corporate decisions, household investment portfolio choices and stock returns. Chen et al. [2]等等）和规范本文参考文献格式。

回应10：感谢审稿专家的意见。已再次修正论文语法和翻译问题，已按照标准格式修改了参考文献格式。

再次感谢审稿专家的宝贵意见。

**Review comments: *major revision***

**Question 1: Can the "lottery culture" of each province from CNRDS be specific to families? Can it be consistent with the family data of CHFS? Please the authors give the answer.**

**Response:** Thank you for pointing this out to us. This paper refers to the practice of Zhao et al. and measures the variable of “lottery culture” by the index obtained from the ratio of per capita lottery sales to per capita GDP of each province. This is an index that represents the “lottery culture” of a province. This paper matches this index with the location of the family in the CHFS database. If the family belongs to this province, then the “lottery culture index” of this province is used to represent the “lottery culture” of the family. We initially intended to achieve city-level data, but as the relevant data on lottery sales at the city level are not published, calculating the lottery culture index at the city level is impossible.

References:

Zhao Q, Zhao W, Lu D, Zhao Q. Lottery and enterprise innovation: A research based on the view of culture. Finance & Trade Economics. 2018; 39:122–40.

Christensen D M, Jones K L, Kenchinton D G. Gambling attitudes and financial misreporting. Contemporary Accounting Research,2018,35(3) ;1229—1261

**Suggestion 1: This paper uses cross-sectional data, so there should be no “area fixed effects” (because this paper is not panel data). Therefore, it is suggested that the author revalidate the empirical results and remove the line "Area fixed effects" to avoid common-sense empirical errors.**

**Response:** Thank you for this suggestion. In the published journals, research on controlling the fixed effects of industries, cities, provinces, and economic regions in cross-sectional data is not limited. For example, Sun Weizeng’s article “*Air pollution and spatial mobility of labor*” was published in *Economic Research Journal* in 2019. In the research design (Y: whether the labor force flows; X: PM2.5), the urban fixed effect is further increased based on the control variables, which alleviates the endogenous problem that may be caused by the omission of urban control variables, obtaining a more robust estimation result.

The “Area fixed effects” used in this paper and the region where the explanatory variables are interpreted are not the same concept. Here, we use the “eastern, northeast, central, and western regions.” The Chinese government divides the provinces into four economic regions according to the level of regional economic and social development: eastern, northeast, central, and western regions. Therefore, this paper also includes the fixed effects of the economic zone into the regression to control regional differences, alleviate the endogenous problems that may be caused by the omission of regional control variables, and obtain more robust estimation results.

**Suggestion 2: It is suggested to add “variable introduction” in this paper, listing “(1) explained variables; (2) Core explanatory variables; (3) Control variables” for more clarity, and should not be mixed into model expressions and data introductions, which appear to be chaotic.**

**Response:** Thank you for this suggestion. In the “3.2 Data Description” section, the titles “3.2.1 interpreted variable,” “3.2.2 core explanatory variable,” and “3.2.3 control variable” are added for clarity.

**Suggestion 3: The third explained variable —— “the diversity of financial portfolio”. The measurement formula is wrong. It is believed that it should not be “****”, but should be “****”，So this is the financial portfolio diversity index.**

Response: We first apologize for this mistake. In the current version of the manuscript, we have corrected it after consulting the literature. Thank you for pointing out this.

**Suggestion 4: It is suggested that the three explained variables in this paper should be abbreviated in the introduction of variables, so as to be consistent with the empirical results in the following article and not make people feel very abrupt.**

**Response:** Thank you very much for sharing this point. To determine the empirical results more clearly in the next parts, this paper will use “Formal” to represent the explained variable “Formal Financial Market Participation,” “Informal” to represent the explained variable “Informational Financial Market Participation,” and “Diversity” to represent the explained variable “Diversity of Financial Portfolio.”

**Suggestion 5: It is suggested that all control variables in the empirical results do not need to be analyzed by empirical results interpretation. In addition, specific regression coefficients and standard errors are not required to be listed in the table of empirical results, and “YES” can be used to represent control, so as to highlight the influence of the core explanatory variables on the explained variables and echo the theme.**

**Response:** Thank you for this great suggestion. According to the suggestion, the control variable is represented by YES, and the interpretation and analysis of the empirical results of all control variables are deleted.

**Suggestion 6: In the explanation of endogeneity, it is suggested to explain in detail the two basic conditions —— exogeneity and correlation conditions —— for the selected instrumental variables (because this paper only explains correlation, not exogeneity);For the instrumental variables method used, the test for weak instrumental variables and the test for exogeneity of instrumental variables (only the weak instrumental variables test is explained in this paper) should be performed, and it is suggested that the authors conduct additional tests to ensure the completeness of the endogeneity explanation in this paper.**

**Response:** Thank you for this great suggestion. This supplement is made in the paper: Whether in theory or reality, sports events do not affect family financial market participation, which is sufficient to show the exogenous nature of instrumental variables. To solve the endogenous problem more rigorously, we conducted the second-stage regression and regress the residual to IV. The p value is greater than 0.1, the original hypothesis is not rejected, and then, IV is exogenous.

**Suggestion 7: Robustness test: For the first two explained variables, this paper uses the logit model to replace the OLS model. For the third explained variable —— the diversity of financial portfolio. Please explain clearly what specific variable is used to replace the third explained variable in this paper (Table 4, Column 5), and suggest that columns 3-4 of Table 4 should be dropped because they have nothing to do with the robustness check for this article.**

**Response:** Thank you for this suggestion. This paper uses the category of household financial assets to measure the diversity of household financial investment portfolios. For example, a household financial investment portfolio includes bonds, stocks, and investment funds. The category of household financial assets is assigned a value of 3 and so on. Table 4 has columns 3–4 to compare the robustness test results with the original empirical results, but there is no problem in deleting them. We deleted these columns following the reviewer’s comments.

**Suggestion 8: In the further analysis, that is, the mechanism test in this paper, the random disturbance terms in equations (3) and (4) should be represented by different letters to distinguish them accordingly; In addition, there is no need to list specific regression coefficients and standard errors for all control variables in the table, and “YES” can be used to represent control.**

**Response:** Thank you for this suggestion. According to the comments, we used ε and replaced μ; the control variable is represented by YES.

**Suggestion 9: It is suggested to add the corresponding research hypothesis of mechanism analysis into the research hypothesis (Hypothesis 3a: "Lottery culture" influences household financial decisions through household risk attitude "; Hypothesis 3b: "Lottery culture" influences household financial decisions through human capital.) In order to echo before and after the part.**

**Response:** Thank you for this suggestion. 3.2. Mechanism analysis has been supplemented.

**Suggestion 10: Pay attention to the translation errors in the text (e.g., Does "Lottery Culture" Affect Household Financial Decisions?—— Evidence from China; Previous research show that "lottery culture" have significant impacts on corporate decisions, Household investment portfolio choices and stock returns. Chen et al.[2], etc.) and standardize the reference format of this paper.**

**Response:** Thank you for this suggestion. The grammar and translation of this paper have been revised again, and the reference format has been modified according to the standard format.

Once again thank you very much for these great comments.

**Response to Reviewer 2**

**Note:** In the review response that follows, we use the font **Arial** to indicate the reviewer’s comments and the font **Times New Roman** to indicate our responses.

**The authors analyze the relationship between “lottery culture” and family financial decision-making through cross-sectional data, and make an analysis of the intermediary mechanism, which is well organized.**

**Response:** Thank you for your appreciation. We truly did our best to improve the manuscript based on your comments.

**However, some issues still need to be improved:**

**(1) What's your definition of “lottery culture”? Can it comprehensively be measured by the ratio of the per capita lottery sales to the per capita GDP of provinces? My suggestion is that the authors should cite more literature.**

**Response:** Thank you very much for sharing these points. This paper has added references to the definition of concepts and variable measures.

After consulting the literature, this paper defines “lottery culture” as reflecting the high degree of recognition of regional decision-makers for “small probability, high yield” events. I made a strengthened explanation in the paper.

At present, most of the papers on lottery culture measured the variable of “lottery culture” using the index obtained from the ratio of per capita lottery sales to per capita GDP of each province, such as Zhao et al. and Christensen et al.

References:

Zhao Q, Zhao W, Lu D, Zhao Q. Lottery and enterprise innovation: A research based on the view of culture. Finance & Trade Economics. 2018; 39:122–40.

Christensen D M，Jones K L, Kenchinton D G. Gambling attitudes and financial misreporting. Contemporary Accounting Research,2018,35(3) ;1229—1261

**(2) Is the result of analysis with cross-sectional data convincing? Why didn’t you use panel data? Please explain why you used cross-sectional data instead of panel data.**

**Response:** Thank you for pointing this out to us. CHFS is a sampling survey project carried out by the China Household Finance Research and Survey Center every year across China. It is a general sampling survey, not a tracking survey. The survey items change every year, making it difficult to form panel data with a unified caliber. In addition, the CHFS survey carried out in 2017 covered 40,011 households in 335 counties in 29 provinces. After deleting the missing items, we had 29,838 observations. The sample size is very large, so the analysis results of cross-section are also representative.

**(3) There are many factors that affect family financial decision-making. Please explain the selection of control variables and the possible reasons for the analysis results.**

**Response:** Thank you for this great suggestion. In the “3.2 Data Description” section, we introduce the selection of control variables and the introduction of control variables in detail. Under the guidance of your valuable opinions, I have made some improvements.

The details are as follows:

Our control variables contain the head of household and household characteristics. Previous studies showed that the head of household and family characteristics will have an important impact on family financial decisions. The head of household is a household member who has the decision-making power. The head of household characteristics include gender, marriage, age, square of age, whether the head of household has medical insurance, whether the head of household has social insurance, health, happiness, and whether the head of household possesses credit cards. The household characteristics contain household income, household debt, and whether the household is located in rural places. Table 1 documents the variable names, descriptions, and sources.

For the result interpretation of control variables, I originally wrote it in “4. Imperial Results.” However, another reviewer suggested deleting this part because he/she believed that control variables were not the main research point of the paper, and control variables should be represented by YES in the empirical results. I temporarily adopted this suggestion. If necessary, I can restore all the contents of the control variables next time.

This is the result interpretation of my control variable: People with social insurance are more likely to participate in the formal financial market and hold more kinds of financial assets but less likely to participate in the informal financial market. As the age grows, people are more willing to participate in the formal financial market and hold more types of assets. However, when the age exceeds a certain threshold, people change their behaviors. People who have credit cards or are in bad health condition are less likely to participate in the financial market and hold less types of assets. People with high income prefer to participate in the financial market and hold diversified financial portfolios, whereas those who have debt prefer to participate in the informal financial market and are less likely to participate in the formal financial market and hold less diversified financial portfolios. Living in a rural region and having happy feelings can impede household participation in the financial market.

**(4) Is there a science to choosing the number of Chinese Football Super League and China Basketball Association teams as the instrument variable? Please explain it by citing more literature.**

**Response:** Thank you for this suggestion.The selection of instrumental variables in this paper refers to the following two studies:

Zhao Q, Zhao W, Lu D, Zhao Q. Lottery and enterprise innovation: A research based on the view of culture. Finance & Trade Economics. 2018; 39:122–40.

Christensen D M, Jones K L, Kenchinton D G. Gambling attitudes and financial misreporting. Contemporary Accounting Research,2018,35(3) ;1229—1261.

**(5) Is there any theoretical support for the intermediary mechanism? Why didn’t you propose the hypotheses on it in part 2 (Hypotheses Development)? My suggestion is that the authors should add some description about mediated relation between “lottery culture” and family financial decision-making in the second part.**

**Response:** Thank you for this suggestion. According to the comments, we have supplemented “2.3 Mechanism analysis” and proposed “Hypothesis 3a. ‘Lottery culture’ influences household financial decisions through risk attitudes” and “Hypothesis 3b. ‘Lottery culture’ influences household financial decisions through human capital.”

Notably, in the “2.1 ‘Lottery Culture’ and Household Participation in The Financial Market,” section, I analyze the theoretical mechanism to determine the causal relationship between “Lottery Culture” and “Household Participation in The Financial Market.” Therefore, Section 2.3 does not discuss too much.

**(6) Is the conclusion of the data analysis in 2017 applicable after 2020 (COVID-19)?**

**Response:** Thank you for giving me a very good research entry point. Under the background of the new crown epidemic, what is the impact of lottery culture on family financial decision-making? Can lottery culture even affect family financial decisions? After the COVID-19 epidemic, some systematic risks have emerged in China’s financial market. The risks of investment and financial management are unprecedented. Do families still have money to invest in the financial market? If so, are they willing to invest in the financial market? This point may be more complex and interesting than the article I am currently discussing. I will go to search and see if there are such data to support this study. I believe it will be very meaningful research.

Once again thank you very much for these great comments.
